# Deoxyribonucleic Acid Methylation in Systemic Lupus Erythematosus: Implications for Future Clinical Practice

**DOI:** 10.3389/fimmu.2018.00875

**Published:** 2018-04-24

**Authors:** Emma Weeding, Amr H. Sawalha

**Affiliations:** ^1^Department of Internal Medicine, University of Michigan, Ann Arbor, MI, United States; ^2^Division of Rheumatology, Department of Internal Medicine, University of Michigan, Ann Arbor, MI, United States; ^3^Center for Computational Medicine and Bioinformatics, University of Michigan, Ann Arbor, MI, United States

**Keywords:** autoimmunity, biomarker, EZH2, IFI44L, lupus, methylation, T cells, therapeutic

## Abstract

Differential deoxyribonucleic acid (DNA) methylation has emerged as a critical feature of systemic lupus erythematosus (SLE). Genome-wide DNA methylation studies have revealed methylation patterns characteristic of SLE—in particular, robust hypomethylation of interferon-regulated genes is a prominent finding in all cells of the immune system studied to date. These patterns reliably distinguish individuals with SLE from healthy controls and from individuals with other autoimmune diseases. For example, hypomethylation within *IFI44L* is both highly sensitive and highly specific for SLE, superior to currently available biomarkers. Furthermore, methylation status of other genetic loci has been associated with clinically relevant features of SLE including disease severity and organ-specific manifestations. Finally, DNA methylation studies have provided important insights into the pathophysiology of SLE. Most recently, there is a growing body of evidence that the transcription factor enhancer of zeste homolog 2 (EZH2) plays an important role in triggering SLE disease activity *via* epigenetic mechanisms, and that EZH2 blockade may be a future treatment option in SLE. In this short review, we discuss the DNA methylation patterns associated with SLE, their relationship to clinically significant features of SLE, and their implications in the development of novel diagnostic and therapeutic approaches to this complex disease.

## Introduction

Systemic lupus erythematosus (SLE) is a highly heterogeneous autoimmune disease that can affect virtually any organ system in the body, resulting in protean clinical and serological manifestations which range from mild to life-threatening. The disease is broadly characterized by the production of antinuclear autoantibodies, resulting in the formation and deposition of immune complexes, which in turn leads to inflammation and damage of affected tissue. As with many other autoimmune diseases, the pathogenesis of SLE is complex and incompletely understood. A genetic component to the disease has long been presumed—first-degree relatives of individuals with SLE have up to a 30-fold higher risk of developing the disease as compared to the general population (1), which clearly suggests some degree of heritability. Indeed, extensive investigation has revealed dozens of genetic risk loci for SLE. Yet, these loci account for less than 20% of disease susceptibility (2). In the vast majority of patients, any one genetic polymorphism in isolation does not confer clinical disease. Rather, SLE arises due to some combination of genetic risk factors and various environmental factors, such as exposure to infections, chemicals, radiation, sex hormones, or other alterations to an individual’s immunologic substrate.

The study of epigenetics has emerged as an important approach in investigating the contributions of both heritable and environmental factors, as well as the interplay between them, in the pathogenesis of autoimmune disease. Epigenetic mechanisms regulate gene expression in a tissue-specific manner by controlling the accessibility of deoxyribonucleic acid (DNA) to the transcription complex without modifying the underlying nucleotide sequence. Epigenetic changes can be either inherited or induced, and can be highly dynamic over the course of a cell’s lifespan. The potentially reversible nature of epigenetic events makes them attractive candidates as biomarkers of disease activity and as targets for therapeutic strategies.

Deoxyribonucleic acid methylation is one of the most well-studied epigenetic mechanisms in humans. Methylation most commonly occurs at the C5 position of cytosine residues in CG dinucleotides and classically results in gene silencing. Conversely, demethylation is generally associated with increased chromatin accessibility and thus active gene expression. Aberrant DNA methylation has emerged as an important epigenetic feature of SLE, and the study of these abnormal methylation patterns has revealed numerous possibilities for a deepened understanding of this complex disease.

In this short review, we discuss the putative role of differential DNA methylation in the pathogenesis and pathophysiology of SLE, with a focus on how these patterns influence clinically relevant features, such as disease severity and heterogeneity, and their implications in the development of novel diagnostic and therapeutic strategies.

## DNA Methylation in the Pathophysiology of SLE

In one of the earliest studies of epigenetic patterns in SLE, Richardson et al. examined the total percentage of methylated cytosine residues in T cells isolated from participants with SLE (3). This revealed significant global DNA hypomethylation of T cells in individuals with SLE when compared to healthy age-matched controls. In a similar vein, the treatment of CD4^+^ T cells by DNA methylation inhibitors, such as procainamide or hydralazine, has been shown to induce hypomethylation and autoreactivity *in vitro* (4). Furthermore, injecting these hypomethylated T cells into syngeneic mice can induce lupus-like autoimmunity *in vivo* (5). While these studies provide a critical foundation in understanding the epigenetic patterns in SLE, the ultimate result of autoreactivity induced *via* hypomethylation is almost a forgone conclusion in that the relationship between DNA methylation inhibitors and autoimmune disease in humans has already been established in clinical practice. Indeed, treatment of humans with procainamide or hydralazine can cause a lupus variant known as drug-induced lupus erythematosus, which has similar clinical manifestations as SLE, and usually resolves within months after discontinuation of the culprit medication.

A seminal work by Javierre et al. was the first to examine genome-wide DNA methylation patterns in SLE and thereby associate epigenetic changes with specific genetic loci (6). Specifically, DNA methylation status was compared in total white blood cells obtained from monozygotic twins who were discordant in SLE status. Global hypomethylation was again observed in SLE participants when compared to their healthy twins or matched controls. Furthermore, sequence-specific demethylation was found in genes associated with several cellular processes which are likely relevant to SLE pathophysiology, including immune response, cell activation, cell proliferation, and cytokine production.

Subsequent studies have examined the DNA methylation patterns of specific cells in the immune system, particularly in T cells, given the earlier evidence of T cell hypomethylation in SLE as described above. In the first study to investigate genome-wide DNA methylation changes in CD4^+^ T cells, the methylation status of over 25,000 CG sites (corresponding to the promoter regions of nearly 15,000 genes) was compared in SLE participants versus healthy controls (7). A total of 341 CG sites were found to be differentially methylated in SLE, with the majority of these being hypomethylated as expected. Hypomethylated genes included *MMP9* and *PDGFRA*, both of which are involved in the development of connective tissue, as well as *CD9*, which has been shown to provide potent costimulatory signals promoting the activation of T cells (8). Hypermethylated genes were primarily involved in metabolic pathways, particularly folate biosynthesis, which is essential in maintaining DNA integrity and stability. Hypermethylation was also noted in *RUNX3*, which encodes a transcription factor that is required for T-cell maturation.

A follow-up study surveyed DNA methylation status across over 485,000 CG sites in naïve CD4^+^ T cells, with the intent to identify methylation changes preceding T cell differentiation and activation, and thereby revealing early epigenetic events which potentially predispose individuals to clinical manifestations of disease (9). A total of 86 differentially methylated CG sites in 47 genes were identified in SLE participants as compared to controls. Most notably, the majority of hypomethylated genes in naïve T cells found in this study are regulated by type I interferons, including *IFIT1, IFIT3, MX1, STAT1, IFI44L, USP18, TRIM22*, and *BST2*. Additionally, gene expression analysis was performed in the same naïve CD4^+^ T cells. This revealed that despite being hypomethylated, none of these interferon-regulated genes were overexpressed in naïve CD4^+^ T cells. Conversely, most of them were significantly overexpressed in total CD4^+^ T cells from participants with SLE. In summary, these results suggest that naïve CD4^+^ T cells undergo epigenetic priming toward a rapid response to type I interferons, resulting in T cell differentiation and activation, and presumably, increased disease activity.

Subsequent studies in memory T cells, regulatory T cells, and neutrophils (including low-density granulocytes) have revealed similar patterns of global hypomethylation, particularly in interferon-regulated genes (10, 11). Most recently, DNA methylation patterns in a T cell subset, specifically CD4^+^CD28^+^KIR^+^CD11a^hi+^ T cells, were examined (12). This previously undescribed subset of T cells has been found to be present in patients with SLE, with the size of the subset correlating to disease severity (13). Differential DNA methylation analysis yet again revealed global hypomethylation in this T cell subset. Moreover, this hypomethylation, in combination with increased chromatin accessibility, resulted in increased expression of pro-inflammatory genes, such as cytokine genes, adhesion molecules, Fc-gamma receptor genes, toll-like receptor genes, human leukocyte antigen molecules, and metalloproteinases. These results further emphasize the important role that this demethylated T cell subset may play in SLE pathophysiology, and suggest that blocking these downstream pro-inflammatory effects might provide novel therapeutic avenues in SLE.

## DNA Methylation as a Biomarker

Even for the experienced physician, SLE can be difficult to diagnose. Owing to the significant heterogeneity of the disease, patients can present with any variety of symptoms at disease onset, some of which may be vague or non-specific. There is no single test for SLE—the diagnosis is ultimately made through clinical judgment by interpreting a patient’s symptomatology in the context of serological, radiographic, and/or histological evidence of disease. Several laboratory markers of SLE are already used in clinical practice, including antinuclear antibodies (ANAs), anti-double stranded DNA (anti-dsDNA) antibody, and anti-Smith (anti-Sm) antibody. Interpretation of autoantibody titers has significant limitations, however. While effectively all patients with SLE test positive for at least one ANA, nearly one-quarter of the general population is also ANA positive (14). Conversely, anti-dsDNA and anti-Sm antibodies are highly specific for SLE, but are only detectable in roughly half of patients (15, 16).

Zhao et al. sought to investigate whether DNA methylation status could act as a more robust biomarker in SLE (17). Specifically, they examined the methylation status of two CG sites located within the *IFI44L* promoter (an interferon-regulated gene found to be hypomethylated in SLE) in DNA from peripheral blood obtained from participants with SLE. They found that a given threshold of hypomethylation at either CG site had a sensitivity and specificity of greater than 90% for SLE versus healthy controls. As discussed above, this is superior to currently available biomarkers such as ANAs or anti-dsDNA antibody. Differential methylation at these CG sites also distinguished SLE from rheumatoid arthritis and primary Sjögren’s syndrome, two autoimmune diseases which can have clinical overlap with SLE. Although *IFI44L* is also hypomethylated in naive CD4^+^ T cells from individuals with primary Sjögren’s syndrome as compared to healthy controls (18), the degree of hypomethylation was found to be significantly higher among those with SLE per the work of Zhao and colleagues. Further validation is required to determine the role of *IFI44L* methylation status in distinguishing these two autoimmune diseases from one another. Nevertheless, the results of this study provide strong evidence that an assay for DNA methylation status in whole blood could be a powerful tool in the diagnosis of SLE. To this point, a summary of the DNA methylation patterns associated with various clinical features of SLE is provided in Table 1. These patterns are discussed in more detail below.

**Table 1:** Summary of differential deoxyribonucleic acid (DNA) methylation patterns in naïve CD4^+^ T cells associated with systemic lupus erythematosus (SLE), disease severity, and organ-specific manifestations.

SLE versus healthy controls
Individuals with SLE exhibit robust DNA methylation changes, primarily hypomethylation, among genes associated with interferon-signaling pathways ▪ Hypomethylation within the *IFI44L* promoter was found to be 94% sensitive and 97% specific for SLE in one study (17) Hypomethylation of interferon-regulated genes is independent of disease activity
**SLE disease activity**
Hypomethylated sites associated with increased disease activity include non-Th1 cytokine genes and human leukocyte antigen class II genes Hypermethylated sites associated with increased disease activity are involved in inhibitory pathways, most notably the transforming growth factor beta signaling pathway Binding sites for the repressive transcription factor enhancer of zeste homolog 2 are enriched among the above hypermethylated loci, and depleted among hypomethylated loci
**Cutaneous SLE**
There is consistent hypomethylation of interferon-regulated genes regardless of cutaneous manifestation (or lack thereof) Unique differentially methylated regions are associated with malar rash, discoid rash, or lack of either Both cutaneous manifestations are uniquely differentially methylated in pathways associated with cell proliferation, apoptosis, and antigen processing and presentation
**Renal involvement**
Individuals with renal involvement exhibit more robust hypomethylation both globally and specifically within interferon-regulated genes compared to those without renal involvement ▪ The type I interferon master regulator gene *IRF7* is only hypomethylated in those with lupus nephritis Hypomethylation within CHST12 is 86% sensitive and 64% specific for lupus nephritis (23)

Systemic lupus erythematosus can be reviewed as a relapsing-remitting disease, with the majority of patients experiencing intermittent flares of disease activity alternating with relative quiescence. Given the evidence of epigenetic T cell priming toward a robust interferon-mediated response, the question arises of whether methylation status might correlate with disease flares. To address this question, Coit et al. performed genome-wide DNA methylation analysis on naïve CD4^+^ T cells from participants with SLE with varying levels of disease activity as measured by SLE Disease Activity Index (SLEDAI) scores (19). They identified over 5,000 CG sites that either negatively or positively correlated with disease activity, and more broadly, discovered that higher disease activity is associated with progressive hypomethylation of genes involved in Th2, Th17, and follicular helper T cell response. Progressive hypermethylation was noted in inhibitory pathways such as the transforming growth factor beta signaling pathway. Gene expression analysis was performed and demonstrated that these epigenetic events do indeed precede gene expression. Overall, these results suggest that not only are naïve CD4^+^ T cells epigenetically predisposed toward an effector T cell response in SLE, but also that these epigenetic changes and putative downstream effects are associated with increased disease activity. The clinical relevance of this conclusion is that determination of DNA methylation status might provide prognostic information in predicting SLE flares, and may thus be useful in tailoring selection of medical therapy and subsequent monitoring of a patient’s response to treatment.

In current clinical practice, selection of therapy for SLE is based not only on overall disease activity but also on a given patient’s particular manifestations of disease. Understanding the methylation patterns of particular manifestations of SLE may provide additional prognostic information and help guide future development of targeted therapies. As an example, cutaneous manifestations such as malar rash or discoid rash are common in patients with SLE. Methylation patterns specific to each of these kinds of rash have been found. Specifically, Renauer et al. compared genome-wide DNA methylation profiles in naïve CD4^+^ T cells from participants with SLE who had a history of malar rash, discoid rash, or neither cutaneous manifestation (20). Between these three groups, they identified several hundred differentially methylated sites, the majority of which were specific to each cutaneous manifestation (or lack thereof). For those with a history of malar rash, the most extensively hypomethylated region was located in the promoter region of precursor microRNA miR-886 (*VTRNA2-1*). Independent studies have shown that hypomethylation of this region modulates signaling pathways which determine cell survival versus apoptosis (21). For those with a history of discoid rash, hypomethylation within *RHOJ* and *HZF* were found, both of which are also involved in determining cell survival versus apoptosis. These results correlate with older findings that the epidermis of patients with cutaneous SLE is in part characterized by an accumulation of apoptotic cells (22).

Another organ commonly affected in SLE is the kidneys. It is estimated that just over half of all patients with SLE have renal involvement, which can range in severity from mild and near-quiescent to fulminant and life-threatening. Early recognition of renal impairment is critical, as treatment is more likely to be successful when it is started as quickly as possible, and conversely, delayed diagnosis is associated with a significantly increased risk of renal failure and death (23). In an effort to determine how DNA methylation patterns might correlate with renal disease in SLE, one study examined genome-wide DNA methylation in naïve CD4^+^ T cells from SLE patients with and without renal involvement (24). The authors discovered 191 differentially methylated CG sites (corresponding to 121 genes) associated with the presence or absence of renal involvement. Genes which were more hypomethylated in SLE participants with renal disease included *IRF7*, which is a well-known genetic risk locus for SLE. Indeed the majority of hypomethylated sites were located in interferon-regulated regions, as expected. Notably, the degree of hypomethylation in these regions was significantly more robust in SLE participants with a history of renal disease, independent of overall disease activity. Genes which were more hypermethylated in SLE participants with renal disease included *CD47*, which has been shown to regulate T cell production of vascular endothelial growth factor (25), and *CD247*, which encodes the T-cell receptor zeta chain, and in turn, plays a key role in antigen receptor-mediated signaling and has been shown to be downregulated in SLE T cells (26). Finally, the authors identified a single CG site, CG10152449 in *CHST12*, for which hypomethylation had a sensitivity of 86% and specificity of 64% in detecting renal disease in SLE participants. No comparable biomarker currently exists in clinical practice.

## DNA Methylation and Future Therapies

Arguably, the ultimate goal in the study of the epigenetics of human disease is not only to identify biological pathways which drive pathophysiology but also to use our new-found understanding of these pathways to develop novel treatment strategies. One of the most promising future treatment options revolves around a transcription regulator known as enhancer of zeste homolog 2 (EZH2). EZH2 is a histone-lysine *N*-methyltransferase enzyme which promotes transcriptional regulation by way of histone methylation as part of the polycomb repressive complex 2. Similar to DNA methylation, posttranslational modifications of histone proteins are epigenetic events which contribute to the pathophysiology of SLE and other autoimmune disorders by regulating gene expression (27). EZH2 trimethylates lysine 27 in histone H3, resulting in H3K27me3 and transcriptional repression. It can also recruit DNA methyltransferases DNMT1, DNMT3A, and DNMT3B (28, 29). When phosphorylated, EZH2 acts as a transcriptional activator at least in part by suppressing H3K27me3, thus disrupting gene silencing (30, 31).

**Figure 1:**
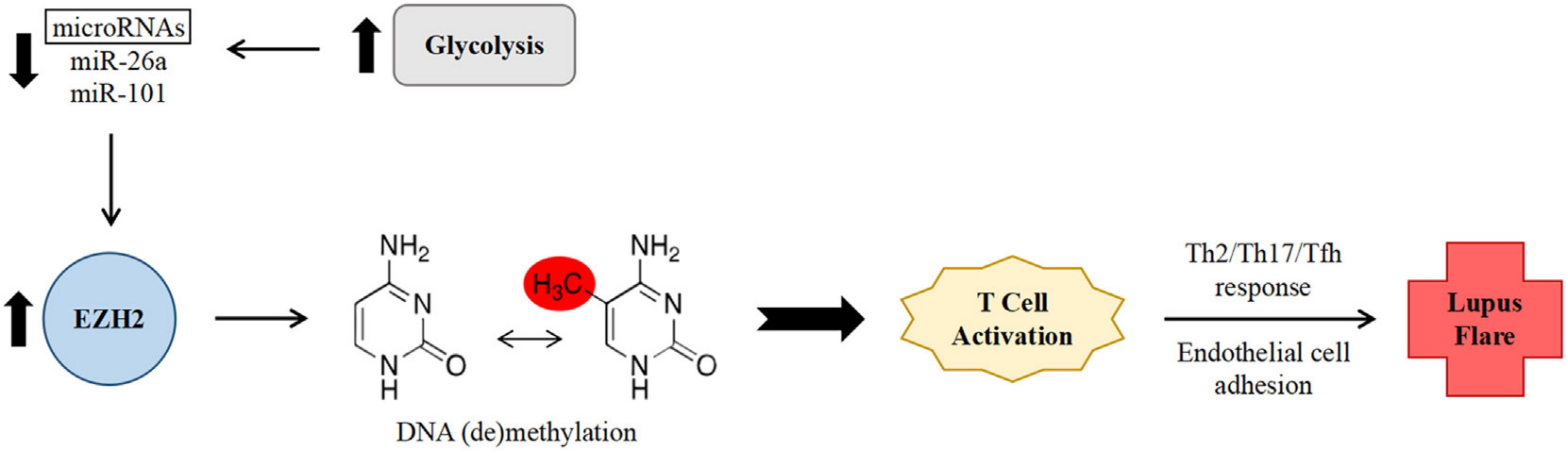
Proposed mechanism of increased systemic lupus erythematosus (SLE) disease activity *via* enhancer of zeste homolog 2 (EZH2)-mediated epigenetic remodeling within CD4^+^ T cells. Abnormally enhanced glycolysis in SLE results in decreased levels of the microRNAs miR-26a and miR-101. Decreased microRNA levels leads to lessened downregulation of the expression of transcription factor EZH2. EZH2 in turn promotes deoxyribonucleic acid methylation changes, leading to T cell activation, a non-Th1 effector T cell response, and increased adhesion to endothelial cells, thereby promoting SLE disease activity.

In the aforementioned study by Coit et al., which examined the relationship between epigenetic changes and disease activity, methylation sites which correlated with disease activity were found to be either enriched (at hypermethylated loci) or depleted (at hypomethylated loci) in binding sites for EZH2, suggesting that it might play an important role in inducing a pro-inflammatory epigenetic shift (19). EZH2 expression in T cells is inhibited by glucose restriction *via* increased expression of microRNAs miR-26a and miR-101 (32). Increased glycolysis has been noted in CD4^+^ T cells from individuals with SLE and in mouse models of lupus, and furthermore, treatment of this abnormally enhanced glycolysis in mice resulted in a shift of immunophenotype toward that of healthy controls (33). As such, it was hypothesized that decreased levels of the above microRNAs, indicating enhanced glycolysis and subsequently increased EZH2 activity, would correlate with increased disease activity in SLE (Figure 1). This association was indeed found when comparing SLEDAI scores to levels of miR-26a expression (19).

Most recently, a follow-up study examined expression levels of EZH2 in CD4^+^ T cells, as well as the effects on DNA methylation associated with EZH2 overexpression, in participants with SLE versus healthy controls (34). First, this study confirmed previous findings that T cell production of EZH2 is downregulated by miR-26a and miR-101. Notably, both of these microRNAs were present at reduced levels in SLE CD4^+^ T cells. Next, overexpression of EZH2 was induced in CD4^+^ T cells from healthy controls, and the resulting genome-wide DNA methylation patterns were assessed. This revealed several hundred differentially methylated CG loci, most notably in regions associated with cell adhesion and leukocyte migration. Indeed, CD4^+^ T cells from both the EZH2-overexpression group and the SLE group showed increased adhesion to human dermal microvascular endothelial cells. Finally, blocking EZH2 effectively reduced the capacity of these T cells to adhere to endothelial cells, providing proof of principle that EZH2 blockade may be a future therapy for SLE. Though no EZH2 inhibitor is yet widely available in clinical settings, one such agent, tazemetostat, is currently being investigated in clinical trials as a treatment for certain cancers.

## Conclusion

Differential DNA methylation has emerged as a critical feature of SLE. Characterization of these methylation patterns has provided important insights into the pathophysiology of this complex disease. Furthermore, assessing an individual’s methylation status shows promise as a future clinical tool and may aid not only in the diagnosis of SLE itself but also act as a prognostic indicator to help predict disease flares and facilitate detection of organ-specific manifestations. Finally, the study of differential DNA methylation and its downstream functional effects on the immunologic environment has now revealed an encouraging potential future treatment option for SLE, namely EZH2 blockade.

## Author Contributions

EW and AS drafted and critically revised the manuscript.

## Conflict of Interest Statement

AS is listed as inventor on a patent application for using IFI44L methylation as a biomarker in SLE. EW declares no conflict of interest. The reviewer HL and handling Editor declared their shared affiliation.

## Abbreviations

*BST2*, bone marrow stromal cell antigen 2; CD, cluster of differentiation; *CHST12*, carbohydrate sulfotransferase 12; DNA, deoxyribonucleic acid; EZH2, enhancer of zeste homolog 2; HLA, human leukocyte antigen; *HZF*, ring finger protein 39; *IFI44L*, interferon-induced protein 44 like; *IFIT*, interferon-induced proteins with tetratricopeptide repeats; *IRF7*, interferon regulatory factor 7; *MMP9*, matrix metallopeptidase 9; *MX1*, MX dynamin like GTPase 1; *PDGFRA*, platelet-derived growth factor receptor A; *RHOJ*, ras homolog family member J; RNA, ribonucleic acid; *RUNX3*, Runt Related Transcription Factor 3; SLE, systemic lupus erythematosus; *STAT1*, signal transducer and activator of transcription 1; TGFB, transforming growth factor beta; *TRIM22*, tripartite motif containing 22; *USP18*, ubiquitin-specific peptidase 18; VEGF, vascular endothelial growth factor; *VTRNA2-1*, vault RNA 2-1.
